# Victim Sensitivity and Its Neural Correlates Among Patients With Major Depressive Disorder

**DOI:** 10.3389/fpsyt.2020.00622

**Published:** 2020-08-11

**Authors:** Xiaoming Wang, Shaojuan Cui, Michael Shengtao Wu, Yun Wang, Qinglin Gao, Yuan Zhou

**Affiliations:** ^1^ Key Laboratory of Behavioral Science, Institute of Psychology, Chinese Academy of Sciences, Beijing, China; ^2^ Department of Psychology, University of Chinese Academy of Sciences, Beijing, China; ^3^ Department of Psychology, Beijing Tongren Hospital, Capital Medical University, Beijing, China; ^4^ School of Sociology and Anthropology, Xiamen University, Xiamen, China; ^5^ The National Clinical Research Center for Mental Disorders & Beijing Key Laboratory of Mental Disorders, Beijing Anding Hospital, Capital Medical University, Beijing, China

**Keywords:** victim sensitivity, major depressive disorder, dorsolateral prefrontal cortex, fractional amplitude of low-frequency fluctuation, resting-state fMRI

## Abstract

**Background:**

Dysfunctional beliefs about the self are common in the development of depressive symptoms, but it remains unclear how depressed patients respond to unfair treatment, both dispositionally and neurally. The present research is an attempt to explore the differences in sensitivity to injustice as a victim and its neural correlates in patients with major depressive disorder (MDD) versus healthy controls.

**Methods:**

First episodic, drug-naïve patients with MDD (*n* = 30) and a control group (*n* = 30) were recruited to compare their differences in victim sensitivity. A second group of patients with MDD (*n* = 23) and their controls (*n* = 28) were recruited to replicate the findings and completed resting-state functional magnetic resonance imaging (fMRI) scanning. Spontaneous brain activity measured by fractional amplitude of low-frequency fluctuation (fALFF) was used to characterize the neural correlates of victim sensitivity both in patients and in healthy controls.

**Results:**

Higher victim sensitivity was consistently found in patients with MDD than healthy controls in both datasets. Multiple regression analysis on the fALFF showed a significant interaction effect between diagnosis and victim sensitivity in the right dorsolateral prefrontal cortex (DLPFC).

**Conclusions:**

The patients with MDD show higher sensitivity to injustice as a victim, which may be independent of their disease course. The MDD patients differ from healthy controls in the neural correlates of victim sensitivity. These findings shed light on the linkage between cognitive control subserved by the DLPFC and negative bias towards the self implicated by higher victim sensitivity among the depressed patients.

## Introduction

Major depressive disorder (MDD) is one of the most common mental disorders, with a high prevalence in current times (1–3). The cognitive model of depression speculates that MDD patients generally hold negative bias and dysfunctional beliefs about themselves. However, it is still unclear how and to what extent self-related personalities and its neural circuits play a role in depressive symptoms (4). Recent research suggests that hypervigilance towards one’s negative experience is linked with the development of MDD (5), and sensitivity to injustice as a victim is related to the stabilization of depressive symptoms (6).

Victim sensitivity captures the individual differences in response to unfair treatment towards oneself. Victim-sensitive individuals tend to detect injustice within a low threshold. Thus, they are likely to experience stronger anger, moral outrage, and exhibit uncooperative behavioral tendencies (7, 8). Victim sensitivity is associated with a higher risk of having negative concerns and social emotions (9) and biased social judgment (for example, people who are higher in victim sensitivity would rate neutral and hostile faces as more untrustworthy and underestimate targets’ cooperativeness in order to avoid being exploited in the future (10)). In the personality space, victim sensitivity has a unique correlation with hostility (11), a facet of neuroticism, which is a predisposition toward depression (12, 13). Furthermore, victim sensitivity was found to be positively related to emotional problems (14) and to mediate the link between attention deficit hyperactivity disorder and depressive symptoms in children and adolescents (14). In a longitudinal study, depressive symptoms at the first measurement predicted higher victim sensitivity one or two years later, while higher victim sensitivity promoted the stabilization of depressive symptoms in those with depressive symptoms at the baseline (6). All of the above findings demonstrate a connection between victim sensitivity and depressive symptoms and also suggest that victim sensitivity should be considered as a potential personality risk factor for the emergence and maintenance of depression. However, there is lack of direct evidence as to whether victim sensitivity was significantly endorsed in a clinical population with MDD, as compared to healthy controls.

We then ask the question: what is the neural basis of higher victim sensitivity in MDD patients? In healthy populations, task-based functional magnetic resonance (fMRI) studies found that the brain activity of anterior insula, anterior cingulate cortex, and dorsolateral prefrontal cortex were increased when participants were faced with unfair treatments (e.g., unequal division on a certain amount of money) (15–17), suggesting that these brain regions may be vital for processing injustice for a victim. Different from the healthy controls, MDD patients had weaker activity in the medial occipital lobe or striatum with increasing or decreasing inequality (18). A relevant study on the neural correlates of justice sensitivity found that victim sensitivity predicted subjective ratings of praise, but it was not correlated with neural activity during a moral judgment task (19). It should be noted that these findings are obtained by using task-based fMRI, in which task-induced brain activity is context-dependent. Personality differences in victim sensitivity may also be reflected in spontaneous brain activity measured by resting-state fMRI, which is a promising tool to uncover the neural basis of inter-individual differences in personality or propensity (20–23). Using an index of spontaneous brain activity that measures temporal synchronization of brain activity within a local brain region, one study found victim sensitivity was positively associated with regional spontaneous brain activity of the paracentral lobule (24). In spite of these descriptions, to our knowledge, no empirical data on the neural basis of victim sensitivity in MDD has been reported. The present study is a first step to fill this gap.

In brief, this current study first aims to test whether the patients with MDD are more sensitive to injustice as a victim. To this aim, we recruited a group of MDD patients who were first episodic and drug-naïve to reduce the potential confounders from long illness duration and medications. Then, we recruited another group of MDD patients with less severe depressive symptoms to test whether the abnormality in victim sensitivity is generalized in patients with MDD. Second, we further explored the neural basis underlying victim sensitivity in MDD using the resting-state fMRI. We selected an index called fractional amplitude of low-frequency fluctuation (fALFF) (25). The fALFF, similar to ALFF, measures low frequency oscillations of the blood oxygen level-dependent (BOLD) signal at rest, but the fALFF represents the ratio of the power spectrum of low-frequency to that of the entire frequency range and thus is less prone to noise compared to ALFF (25). The low frequency BOLD oscillation are closely related to spontaneous neuronal activity (26). As a method with high test-retest reliability to detect the intensity of regional spontaneous neuronal activity during rest (27), the fALFF has been widely used to investigate spontaneous brain activities in different psychiatric disorders, including MDD (28–30), and be linked with personality traits in healthy populations (31–33). In addition, fALFF is a data-driven approach and requires no *a priori* hypothesis and thus is appropriate for exploratory analyses, such as the present study. Therefore, here we attempted to employ fALFF to explore whether there are different neural correlates of victim sensitivity between patients with MDD and healthy controls.

## Methods

### Participants

Two groups of patients with MDD and their matched healthy controls were recruited from two independent sites. Participants in the Tongren dataset were recruited for the aim of investigating whether MDD patients who are first episodic and drug-naïve are more sensitive to injustice as a victim on a behavioral level. We then used another group of participants (i.e. Anding dataset) to test whether the findings obtained in the Tongren dataset can be replicated in MDD patients with less severe depressive symptoms. The participants in the Anding dataset are from a project that initially aimed to study the neural basis of abnormal social decision-making behavior and related traits in patients with MDD, and thus made it possible to further investigate the differences in neural correlates of victim sensitivity among MDD patients versus healthy participants in the current study.

In the Tongren dataset, 30 patients with MDD were recruited from the Department of Psychology, Beijing Tongren Hospital, Capital Medical University. Thirty demographically-matched healthy controls were recruited *via* advertisements. All of these patients met the following criteria: (1) the Diagnostic and Statistical Manual of Mental Disorders, fourth edition (DSM-IV) criteria for a major depressive episode, diagnosed independently by two qualified psychiatrists who interviewed the patients personally; (2) seeking medical advice for the first time in this hospital and have never taken any psychotropic medication; (3) Hamilton Rating Scale for Depression (HAMD-17) scores >=17; and (4) age range is between 18 and 45 years old. The patients were excluded if they had any preexisting or concurrent co-morbid primary diagnosis that met the DSM-IV criteria for any Axis I disorder other than MDD. Participants in the control group had no current or past history of depression or any other psychiatric disorders and no family history of major psychiatric or neurological illness in first-degree relatives. Additional exclusion criteria for both of the groups were acutely suicidal or homicidal behaviors, history of trauma resulting in loss of consciousness, history of major neurological or physical disorders that could lead to an altered mental state, or current pregnancy or breastfeeding.

In the Anding dataset, 23 patients were recruited from Beijing Anding Hospital, Capital Medical University. Potential applicants for the patient group were from the outpatient departments. They were recommended by their doctors who had diagnosed them before, and then again diagnosed by a psychiatrist when they were recruited in this study. The inclusion and exclusion criteria of the patients were the same as the Tongren dataset, with the only exception that these patients were not experiencing an acute depressive episode and had less severity in depressive symptoms (for details, see **Table 1**). Among these MDD patients, 15 patients had at least two onsets of depressive episodes; eight patients had a recent depressive episode and their symptoms had been controlled after antidepressant treatments when they were recruitment. Only one patient did not take any psychotropic medication; the other patients received antidepressant medications (SSRIs and/or SNRIs), with one patient additionally taking low-dose benzodiazepines. Twenty-eight healthy controls were recruited by advertisements. Due to the relatively stable mental condition of the MDD patients in the Anding dataset, only participants in the Anding dataset participated in MRI scanning.

**Table 1:** Demographics and clinical information of patients with MDD and healthy controls within the two datasets.

Characteristics	Tongren dataset			Anding dataset		
	MDD	NC	p^a^	MDD	NC	p^a^
Sample size	N=30	N=30		N=23	N=28	
Age (years)	25.47 ± 6.40	27.67 ± 4.14	0.12	31.22 ± 5.70	29.57 ± 5.80	0.32
Gender (M: F)	6:24	6:24	1.00	13:10	16:12	0.96
Education	14.33 ± 2.44	14.80 ± 2.19	0.44	14.91 ± 3.22	15.25 ± 2.55	0.68
HAMA	22.03 ± 4.92	–	–	9.83 ± 6.86	0.64 ± 1.16	<0.001
HAMD-17	24.90 ± 4.14	–	–	13.13 ± 6.96	0.89 ± 1.72	<0.001
Victim Sensitivity	34.67 ± 9.06	25.10 ± 7.48	<0.001	30.96 ± 9.50	25.41 ± 8.07	0.03

a Two-sample t-tests or chi-square test. HAMD-17, the Hamilton Rating Scale for Depression; HAMA, the Hamilton Rating Scale for Anxiety.

This study was approved by the Institutional Review Boards of Beijing Tongren Hospital, Beijing Anding Hospital, Capital Medical University, as well as the Institute of Psychology, Chinese Academy of Sciences. Written informed consent was obtained from all participants.

### Assessment of Victim Sensitivity

Ten items were used to measure justice sensitivity as a victim (11, 34). Examples of items for victim sensitivity are “It makes me angry when I am treated worse than others” or “ I ruminate for a long time when other people are treated better than me”. Each item was scored on a 6-point rating scale ranging from 0 (not at all) to 5 (exactly). The reliabilities were acceptable with the estimated reliability coefficients (Cronbach’s alpha) of the MDD patients and healthy controls amounting to 0.92 and 0.84 for the Tongren sample, and to 0.84 and 0.89 for the Anding sample.

### MRI Data Acquisition

The fMRI data were acquired using a GE 3.0 T MRI scanner at the Magnetic Resonance Imaging Research Center, Institute of Psychology, Chinese Academy of Sciences. Structural and functional MRI scans were obtained for each participant. Structural MRI scans were acquired using a magnetization-prepared rapid acquisition gradient echo sequence with the following parameters: TI = 450 ms, receiver bandwidth = 31.25, matrix = 256 × 256, field of view (FOV) = 240 mm * 240 mm, slice thickness = 1.5 mm, flip angle = 12°. Functional images were acquired for each participant using an echo planar imaging (EPI) sequence with the following parameters: TR = 2000 ms, TE = 30 ms, flip angle = 70°, acquisition matrix = 64 * 64, and FOV = 220 mm * 220 mm; 33 axial slices, with a thickness of 4 mm and no gap. During the scanning, the participants were instructed to keep their eyes closed and not to focus their thoughts on anything. The duration of the resting-state fMRI was 10 minutes. After resting-state fMRI scanning, these participants performed a fMRI task, but the data were not used in the current study.

### Image Preprocessing

Conventional functional imaging preprocessing was performed using the Data Processing Assistant for Resting-State fMRI (DPARSF 4.3, http://rfmri.org/dpabi) (35), which is based on Statistical Parametric Mapping (SPM12) (http://www.fil.ion.ucl.ac.uk/spm) and the Resting-State fMRI Data Analysis Toolkit (REST 1.8, http://www.restfmri.net) (36). Conventional preprocessing steps were conducted, including the removal of the first 5 volumes, slice timing realignment, co-registration, segmentation of the T1 map to generate the gray matter (GM), white matter (WM), and cerebrospinal fluid (CSF) (37), nuisance variable regression, spatial normalization with 2-mm cubic voxels, and spatial smoothing of 4 mm FWHM. The nuisance variables included 24 motion parameters (6 head motion parameters, 6 head motion parameters one time point before, and the 12 corresponding squared items), 5 principal components from the individual segmented CSF and WM regions (38), and linear and quadratic trends (39). To quantify head motion, the volume-based framewise displacement (FD) was computed (40).

### fALFF Analysis

For each participant, we calculated the fALFF to characterize the regional spontaneous activity in a voxel-wise way using the DPARSF software. The fALFF is defined as the total power within a frequency band divided by the total power of the entire detectable frequency range (25). Thus, the square root was firstly calculated at each frequency in the power spectrum, and the averaged square root was computed across a low frequency range (0.01–0.08 Hz) at each voxel. Then, the fALFF was obtained as a fraction, which was the sum of the amplitude across 0.01–0.08 Hz divided by that across the whole frequency range. Finally, the normalized score of fALFF was obtained and an individual voxel-wise z-fALFF map was generated for each participant.

### Statistical Analysis

Chi-square tests and independent sample t tests were conducted to compare the mean level of demographic, clinical variables, and victim sensitivity between the MDD and healthy controls for each site. Correlation analyses and partial correlation analyses were conducted to investigate the clinical correlates of victim sensitivity for each dataset. All of these analyses were conducted by SPSS v21.

To identify the brain regions where activities related to victim sensitivity may be different between patients with MDD and healthy controls, we firstly conducted a multiple regression analysis on the z-fALFF images of the total sample using SPM12. The z-fALFF of each voxel in the brain were regressed on the variables of diagnosis, victim sensitivity score, and the diagnosis by victim sensitivity score interaction, with age, gender, education level, and mean FD as covariates. To control for Type 1 error, a cluster-level family-wise error (FWE) corrected *p*-value of 0.05 was used for multiple comparisons correction (individual voxel height threshold of *p* < 0.001). Then, multiple regression analyses were conducted on the z-fALFF images for patients and controls, separately. The victim sensitivity score was entered as a regressor into the model, in addition to age, gender, education level, and mean FD which were included as variables of no interest. Due to the relatively small sample size, we conducted a Small Volume Correction (SVC) by using the result of the regression analysis of the total sample as a mask (individual voxel height threshold of *p* < 0.005, cluster-level FWE corrected *p* < 0.05 following SVC).

## Results

### Demographic and Clinical Data

There were no significant differences in gender composition, age, and educational level between patients and healthy controls in both of the two datasets (all *p* > 0.05) (**Table 1**). The patients in the Tongren dataset showed more severe depressive symptoms as assessed with the HAMD (p<0.001) and more severe anxiety symptoms as assessed with the Hamilton Rating Scale for Anxiety (HAMA) (p<0.001) compared with those in the Anding dataset.

### Victim Sensitivity

We found higher victim sensitivity among MDD patients both in the Tongren dataset (MDD: 34.67 ± 9.06, NC: 25.10 ± 7.48; p<0.001, Cohen’s d = 1.15) and in the Anding dataset (MDD: 30.96 ± 9.50, NC: 25.41 ± 8.07; p=0.03, Cohen’s d = 0.63) (**Table 1**).

We also explored the clinical correlates of victim sensitivity. We found that there was no correlation between victim sensitivity and the HAMD score in the Tongren dataset (*r* = 0.13, *p* = 0.49) but positive correlation in the Anding dataset (*r* = 0.54, *p* = 0.007). However, the correlation in the Anding dataset was marginally significant after controlling for the HAMA score (*r* = 0.42, *p* = 0.053) or disappeared after excluding the remitted patients (HAMD score < =7; N=6) (*r* = 0.05, *p* = 0.86). All of these findings suggested that the correlation between victim sensitivity and the severity of depressive symptoms may not be reliable.

### fALFF

**Figure 1:**
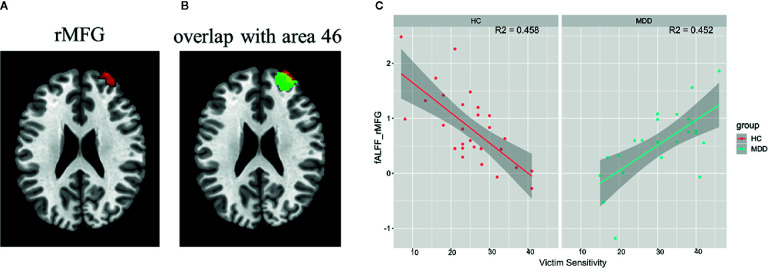
Interaction effect between diagnosis and victim sensitivity on fALFF and the results of post hoc analysis. **(A)** The region showing significant interaction effect between diagnosis and victim sensitivity; **(B)** The region showing significant interaction effect was overlaid with the area 46 in the Brainnetome Atlas Reviewer (http://atlas.brainnetome.org/). **(C)** Scatter plots for the relationship between the fALFF value and victim sensitivity within each group with the shadow parts representing 95% confidence interval. Abbreviation: rMFG, right middle frontal gyrus.

A significant interaction effect between diagnosis and victim sensitivity was found in the right middle frontal gyrus (peak MNI coordinate: [34, 58, 28]; cluster size: 95 voxels; cluster-level FWE *p* < 0.05) (**Figure 1A**). By searching this region in the Brainnetome Atlas Viewer (41), we found that this region was located in the area 46, which is often termed as the dorsolateral prefrontal cortex (DLPFC) (**Figure 1B**). The interaction effect was shown in **Figure 1C**, which illustrated the relationship between victim sensitivity and the z-fALFF value in the right the DLPFC for each group.

By separately analyzing the relationship between victim sensitivity and fALFF within each group, we found that there were no regions showing significant correlations with victim sensitivity score both in the MDD group and in the healthy controls group in a whole brain search (cluster-level FWE *p* < 0.05). But by using SVC with the results of the regression analysis of the total sample as a mask, we found a significantly negative correlation in the right DLPFC in the healthy controls group (SVC cluster-level FWE corrected *p* = 0.01, 26 voxels, T = -4.45, peak voxel coordinates: [32, 56, 26]) and significantly positive correlation in the right DLPFC in the MDD group (SVC cluster-level FWE corrected *p* = 0.04, 9 voxels, T = 3.35, peak voxel coordinates: [32, 58, 22]).

**Figure 2:**
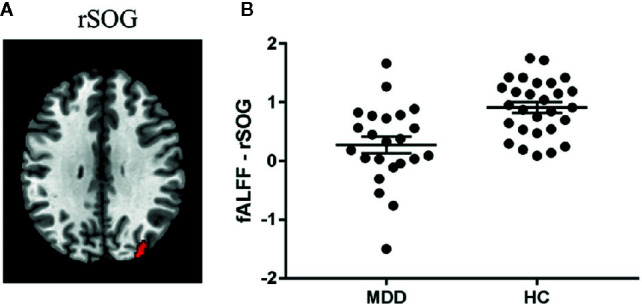
Diagnosis effect on fALFF. **(A)** Region showing significant group difference in fALFF between the patients with major depressive disorder (MDD) and the healthy controls (HC). **(B)** The fALFF value in this region within each group. Abbreviation: rSOG, right superior occipital gyrus.

Significant diagnosis effect was found in the right superior occipital gyrus (SOG; peak MNI coordinate: [32, -84, 42]; cluster size: 104 voxels) (**Figure 2**), suggesting that the patients showed decreased fALFF in this region compared to the healthy controls. We didn’t find the regions showing significant correlations with victim sensitivity across the two groups in the pre-specified threshold (cluster-level FWE *p* < 0.05).

## Discussion

The current study provides robust evidence that MDD patients have higher sensitivity to injustice as a victim, which was consistently shown in two clinical samples. The results also showed that the neural correlates of victim sensitivity differ between the MDD patients and the healthy controls. Specifically, the fALFF in the right DLPFC, the region for cognitive control, positively correlated with victim sensitivity in MDD patients, but was negatively correlated with victim sensitivity in healthy controls.

### Hypersensitivity to Injustice as a Victim

In line with previous studies on the association between victim sensitivity and depressive symptoms in non-clinical samples or personality traits related to depression (6, 42, 43), the present study found that patients with MDD had higher sensitivity to injustice as a victim. Particularly, this hypersensitivity can be found in a group of first episodic, drug-naïve patients with MDD, suggesting that the increased victim sensitivity was free from the confounding effect of chronic illness or antipsychotic drugs and could be observed even in the early stage of depression. In order to test whether this hypersensitivity can be generalized to a common clinical sample, we recruited another group of MDD patients, most of whom were medicated and with mild to moderate depressive symptoms (the Anding dataset). We again found increased victim sensitivity in these patients. On the contrary, we found the correlation between victim sensitivity and severity of depressive symptoms was not reliable because we only found the evidence that supported a positive correlation in the Anding dataset not in the Tongren dataset. More importantly, the correlation only showed the trend towards significance after controlling for the anxiety symptoms or disappeared after excluding the remitted patients who had extremely low HAMD scores. The non-repeatable correlation between victim sensitivity and severity of depressive symptoms suggests that the increased victim sensitivity may be independent of depressive severity; however, this conclusion should be verified with a larger sample size and in the remitted patients. All of these findings - the repeatable findings on the higher victim sensitivity in the MDD patients in both of the two sites and the non-repeatable clinical correlates of the victim sensitivity - suggest that higher victim sensitivity may be independent of illness course, severity, or medication. Combining the previous findings obtained in non-depressed samples with ours, it seems higher victim sensitivity may be an important psychopathological feature for depression. In a longitudinal study of German adolescents, victim sensitivity increased the stabilization of depressive symptoms if they were present at baseline, suggesting that victim sensitivity may be a maintaining factor for depression (6). In future work, the role of victim sensitivity in the occurrence and progression of depression should be further examined in a longitudinal study.

The present study indicated that patients with MDD are more likely to perceive injustice and respond to injustice to one’s own disadvantage. Negative bias and dysfunctional beliefs about the self may account for this hypersensitivity in MDD patients. Much evidence supports the notion that MDD patients may suffer from dysfunctional cognition, which leads them to generate negative perceptions of social interactions in line with the cognitive model of depression (4). A negative bias has repeatedly been observed among MDD patients when processing facial emotions and moral and social emotions (for a review, please see 44). Dysfunctional beliefs about the self are also observed in MDD. For example, the patients with MDD showed increased affective responses to the same events as the healthy controls experience, as observed in the patients with chronic depression when they were facing mood induction materials individualized with autobiographical content (45). The MDD patients tended to judge a proposal as less fair when they are in disadvantaged situation (46), suggesting they were more sensitive to injustice. These patients were more likely to reject an unfair proposal as retaliation (46, 47). Therefore, it is possible that this negative bias and dysfunctional cognition about the self makes MDD patients more sensitive to injustice. Finally, we should note that depressive symptoms often lead to deterioration of social relationships and vice versa (48, 49), which will increase the likelihood of exposure to a disadvantaged situation and thus increase the chance of experiencing injustice as a victim.

### Neural Basis of Increased Victim Sensitivity

Using fALFF as a search tool, we found a contrary pattern regarding the association between the spontaneous activity in the right DLPFC and victim sensitivity among the MDD patients versus healthy controls. Specifically, for healthy populations, the level of spontaneous activity in the right DLPFC was negatively correlated with the level of victim sensitivity. However, for MDD patients, the level of spontaneous activity in the right DLPFC was positively correlated with the level of victim sensitivity.

The right DLPFC is strongly associated with cognitive control (50, 51). It is well-established that superior cognitive control is related to lower levels of unwanted emotional responses, such as anger and aggression (52). A temporal decrease in the activity of the right DLPFC using neuromodulation techniques increased revenge-seeking, emotion-driven behavior (53), supporting the role of cognitive control subserved by the right DLPFC in overcoming unwanted emotional responses. Further evidence for the role of cognitive control in victim sensitivity is seen in the relationship between decreased spontaneous activity in the DLPFC indicated by regional homogeneity and higher rumination in healthy subjects, which suggests less efficient inhibitory control on the increased negative self-focused conflicts (54). Taking all of this evidence together, it is possible that individuals with low sensitivity to injustice as a victim have already had efficient control resources to self-regulate the influence of the disadvantaged situations (i.e., less likely to respond to injustice to one’s own disadvantage *via* anger, sadness, and rumination), and this effortful self-control is reflected by the higher spontaneous activity in the right DLPFC. We noted that this finding is in contrast to a previous study, which found that victim sensitivity was positively correlated with regional activity of the paracentral lobule (24). This inconsistency might be due to the differences in the index measuring spontaneous brain activity. Different from the fALFF, which reflects the spontaneous brain activity in an area (25), used in the present study, regional homogeneity was used in Wu’s paper, which measures the temporal synchronization of the time series of an area’s nearest neighbors (55).

It’s interesting that, unlike the healthy controls, the MDD patients revealed a positive correlation between victim sensitivity and the fALFF in the right DLPFC. This reverse pattern between depressed patients and healthy controls is also seen in previous studies, such as the relationship between the DLPFC volume and rumination (54). MDD is characterized by a failure in cognitive control of emotion (56). Although many studies found hypoactivity in the DLPFC in the MDD patients, increased or unchanged brain activity in this region compared to the healthy controls are also reported. For example, increased activity in the DLPFC to negative stimuli has been consistently found in young MDD patients based on a meta-analysis, suggesting that MDD patients have inefficient emotional regulation during affective processing (57). MDD patients also showed increased brain activity in the DLPFC to maintain a similar level of behavioral performance as controls during cognitive control (58). The hyperactivity in the DLPFC may be a reflection of inefficient cognitive control or compensation in the MDD patients. It needs to be noted that in this study we found that the spontaneous activity in the right DLPFC in the MDD patients was comparable with that in the healthy controls, but it was positively correlated with victim sensitivity in the patients. This finding may suggest that, even though the MDD patients recruit cognitive control resources to regulate their emotional responses (e.g., anger or rumination) when they are in an unjust situation, they fail in inhibiting the unwanted emotions or ruminating on one’s own disadvantage due to inefficient cognitive control or failure in recruiting more resources, and are thus more likely to respond to unjust situations *via* emotional responses (i.e., high sensitivity to injustice as a victim).

The cognitive control explanation subserved by the DLPFC is compatible with negative bias, which may be the candidate psychological process behind the hypersensitivity in the MDD patients as we discussed. Previous studies on the neural basis of negative bias have already indicated that impaired top-down cognitive control reflected by deficits in the DLPFC function play a vital role in negative bias (59). However, the exact psychological mechanisms still need to be determined in the future.

### Limitations

There are several possible limitations in this study. First, even though higher victim sensitivity was found in both of the two samples, the neural basis of higher victim sensitivity in the MDD patients was only examined in one sample, some of whom were in the remission period and most of whom were medicated. Previous studies suggest differences in spontaneous brain activities and connectivity between MDD patients experiencing their first episode and remitted MDD patients or between drug-naïve MDD patients and medicated MDD patients (29, 60, 61). Thus, further validation is needed in a group of patients with higher homogeneity in clinical profiles, such as a group of patients who are experiencing their first episode and are medication free, or a group of remitted patients. It is more interesting to investigate whether the correlations between victim sensitivity and spontaneous brain activities is trait- or state-related. Secondly, the sample size is relatively small in the current study, especially in the part of neuroimaging analysis; however it is comparable to previous studies that involve the recruitment of MDD patients for fALFF analyses (for a review, please see also 29). The current findings need to be validated using a larger sample size. Thirdly, our findings suggest that the right DLPFC plays a significant role in victim sensitivity, both in the healthy controls and in depressed patients; however, the exact function of the DLPFC in victim sensitivity still needs to be determined by the adoption of task-based fMRI. For example, in an ultimatum game, increased activity in the right DLPFC has been repeatedly observed when the participants faced unfair proposals or unjust treatment (15, 17).

## Conclusions

In summary, the current study investigated the hypothesis that the MDD patients have higher victim sensitivity and obtained consistent findings across two clinical samples. Furthermore, we found that the MDD patients differ from the healthy controls in the neural correlates of victim sensitivity. That is, the spontaneous activity in the right DLPFC showed contrary correlation with victim sensitivity in the healthy controls and in the MDD patients. These findings enrich our understanding of personality traits related to depression, and shed light on the link between cognitive control and negative bias about the self among MDD patients.

## Data Availability Statement

The datasets generated for this study are available on request to the corresponding author.

## Ethics Statement

The studies involving human participants were reviewed and approved by Institutional Review Boards of Beijing Tongren Hospital, Beijing Anding Hospital, Capital Medical University, as well as the Institute of Psychology, Chinese Academy of Sciences. The patients/participants provided their written informed consent to participate in this study.

## Author Contributions

All authors contributed to the article and approved the submitted version. SC and QG collected data. XW, SC, and YW analyzed the data. YZ designed this study. XW, YZ, and MW drafted the manuscript. All authors revised the manuscript.

## Funding 

Funding for this study was provided by the Natural Science Foundation of China (grant number 81771473) and the State High-Tech Development Plan of China (863) (grant number 2015AA020513), which provided support for the data collection.

## Conflict of Interest

The authors declare that the research was conducted in the absence of any commercial or financial relationships that could be construed as a potential conflict of interest.
